# “If the social circle is engaged, more pregnant women will successfully quit smoking”: a qualitative study of the experiences of midwives in the Netherlands with smoking cessation care

**DOI:** 10.1186/s12913-022-08472-7

**Published:** 2022-08-31

**Authors:** Eefje Willemse, Bethany Hipple Walters, Linda Springvloet, Jeroen Bommelé, Marc C. Willemsen

**Affiliations:** 1 The Netherlands Expertise Centre for Tobacco Control, Trimbos Institute, Da Costakade, 45 3521 VS Utrecht, The Netherlands; 2 Massachusetts General Hospital, Boston, USA; 3 Department of Health Promotion, Maastricht University, Maastricht, The Netherlands

**Keywords:** Midwives, Smoking cessation counselling, Pregnancy, Partners, Social circle, Qualitative research

## Abstract

**Background:**

If smoking is common within a pregnant woman’s social circle, she is more likely to smoke and her chances of succeeding in quitting smoking are reduced. It is therefore important to encourage smoking cessation in a pregnant woman’s social circle. Midwives are ideally positioned to help pregnant women and members of their social circle quit smoking but there is currently little knowledge about if and how midwives approach smoking cessation with pregnant women’s social circles.

**Methods:**

In 2017 and 2018, semi-structured interviews were conducted with 14 birth care providers in the Netherlands. Interviews were inductively coded; data were analyzed thematically.

**Results:**

In the interviews, midwives reported that they don’t commonly provide smoking cessation support to members of pregnant women’s social circles. The respondents noted that they primarily focused on mothers and weren’t always convinced that advising the partners, family, and friends of pregnant women to quit smoking was their responsibility. Data from the interviews revealed that barriers to giving advice to the social circle included a lack of a trusting relationship with the social circle, concerns about raising the topic and giving unwanted advice on cessation to members of the social circle and a lack of opportunity to discuss smoking.

**Conclusions:**

Midwives in the Netherlands were reluctant to actively provide smoking cessation advice to the social circle of pregnant women. To overcome barriers to addressing cessation to the social circle, educational programs or new modules for existing programs could be used to improve skills related to discussing smoking. Clear guidelines and protocols on the role of midwives in providing cessation support to the social circle could help midwives overcome ambivalence that they might have.

**Supplementary Information:**

The online version contains supplementary material available at 10.1186/s12913-022-08472-7.

## Background

Maternal tobacco smoking is a major modifiable risk factor for perinatal health problems [1]. Smoking during pregnancy can cause adverse maternal and fetal health outcomes, including stillbirth [1], miscarriage [2], premature birth [3, 4], placental abruption [5], and low birth weight [3, 4]. Infants exposed to tobacco smoke are at greater risk for sudden infant death syndrome [1], childhood obesity [6, 7], and ear infections and upper respiratory infections [8]. These risks can be reduced by helping pregnant women quit smoking and helping them reduce exposure to tobacco smoke [9].

Pregnancy is an opportunity for pregnant women and people in their social circle to change their health behavior, such as quitting smoking [10, 11]. However, not all women quit smoking during their pregnancy. In Europe, the estimated prevalence of smoking during pregnancy is around 8% [12]; 30% of women who smoked before pregnancy continued to smoke daily during pregnancy [12]. In 2018, 7% of pregnant women in the Netherlands smoked at some point during pregnancy; 23% of the women who smoked before pregnancy continued smoking during the entire pregnancy [13].

A pregnant woman’s partner, friends, and family (her ‘social circle’) were found to be a major determinant for maternal smoking cessation during pregnancy [14]. Research shows that a pregnant woman’s social circle directly influences her smoking through the support they give and by changing their own smoking behavior [15–18]. However, if a pregnant woman lives with people who smoke and if smoking is common within her social circle, her chances of succeeding in quitting smoking have been shown to be reduced [19]. In addition, exposure to second and third hand smoke is associated with adverse health effects for mothers and children [4, 20, 21]. Healthcare providers can be effective at assisting members of a pregnant woman’s social circle in quitting or encouraging them not to smoke near a pregnant woman [22].

Due to the harms and risks to maternal and fetal health associated with tobacco use and tobacco smoke exposure [4, 20, 21], it is important that obstetrician-gynecologists (OB-GYNs) and midwives (hereafter birth care providers) address tobacco use and tobacco smoke exposure with the pregnant women’s social circle. Pregnancy can serve as a teachable moment for smoking cessation for a pregnant woman’s social circle, particularly partners, and an opportunity for birth care providers to help the pregnant woman and members of her social circle to quit smoking [23–25]. Engaging the social circle has the potential to increase the effectiveness of smoking cessation programs for pregnant women [8] and can improve the public health impact of these programs by increasing the number of people who quit smoking. However, the members of pregnant women’s social circle often receive little to no smoking cessation advice or assistance from birth care providers [26].

In the Netherlands, midwives are the primary birth care providers for the majority of pregnant women. In accordance with the Netherlands Healthcare Inspectorate regulations, midwives should counsel pregnant women and their partners about quitting smoking using the V-MIS protocol (Minimale Interventiestrategie Stoppen met Roken voor de Verloskundigenpraktijk – Minimal Intervention Smoking Cessation Strategy for Midwifery Practices) [27, 28]. The protocol consists of asking pregnant women and their partners about smoking, working to increase motivation to quit, addressing barriers to cessation, setting a quit date, discussing cessation tools and techniques, offering help after the quit date, and working to prevent relapse [27]. Research on the use of the protocol also showed that 81% of midwives almost always learn the smoking status of pregnant women’s partners, yet midwives lacked skills in motivational interviewing [29] and didn’t follow all of the steps of the V-MIS protocol [30]. Further research is needed on the provision of smoking cessation support by birth care providers to the social circle of pregnant women.

### Aims

The aim of this study is to explore, through interviews, experiences of birth care providers in the Netherlands with providing smoking cessation support to members of pregnant women’s social circle.

The results of this study provide insight into the ways in which birth care providers assist members of pregnant women’s social circle with smoking cessation, as well as into the barriers that birth care providers have faced when working with a pregnant woman’s social circle. Data from this study can be used by health policy developers, birth care educators, and birth care providers to further develop, improve, and implement smoking cessation care guidelines for midwives, especially guidelines in the Netherlands or in healthcare systems with a similar structure of midwife-delivered care for pregnant women.

## Methods

### Birth care in the Netherlands

In the Netherlands, midwives are trained in prenatal care, birth care, and postnatal care through a 4 year direct-entry Bachelor of Science in Midwifery program [31]. After completing their education, midwives register as healthcare professionals with the Netherlands Ministry of Health, Welfare and Sport. In 2016, 3221 midwives were registered healthcare professionals in the Netherlands, with the majority working in primary care midwifery practices [32].

Midwifery care for pregnant women begins in the eighth week of pregnancy and continues until a few weeks after birth [31]. Most pregnancies in the Netherlands are, at least at first, cared for by primary care midwives who practice in independent midwifery practices. If complications arise during pregnancy, midwifes refer women to hospital-based care, where care is provided by an OB-GYN or secondary care midwife for as long as necessary.

### Design of data collection

In order to understand birth care providers’ experiences with providing smoking cessation support to the social circle of pregnant women, semi-structured interviews were conducted with fourteen birth care providers in the Netherlands. The interviews were conducted using an interview guide. The interview guide included an introduction to the interview, a description of the goals of the interview, and questions about birth care providers’ experiences with addressing tobacco use and exposure with the social circle of pregnant women, barriers in discussing cessation with the social circle, and the role of birth care providers in cessation support to the social circle. An English version of the interview guide is provided in Additional File 1.

### Ethical approval

The research was approved by the Trimbos Ethics Committee in October 2017 (2362208) and was carried out in accordance with the 1964 Helsinki Declaration and its later amendments. The data presented in this article comes solely from the interviews with birth care providers and does not include any data from patients or any identifiable data about patients or members of patients’ social circles.

Prior to interviews, all participants signed a consent form stating that they were informed that participation was voluntary, that they could withdraw at any time, that they were willing for the interview to be recorded, and that the data would be analyzed anonymously. No incentives for participation in the interviews were offered.

The audio files and transcripts were saved on a secured drive and were only accessible to those analyzing the data (EW and LS). All data from the interviews has been presented anonymously.

### Participant selection and recruitment

The study team aimed to conduct at least twelve interviews with birth care providers; this number is in line with guidance on interviews and saturation, which notes that 10–12 interviews are often sufficient for reaching data saturation [33, 34]. The interviews and the data analysis were conducted simultaneously. We reached saturation at interview 11. However, two additional interviews were conducted to ensure no new information emerged.

A mix of purposeful sampling and convenience sampling was used to recruit birth care providers for interviews. First purposeful sampling was used to engage with birth care providers working often with people who smoke. Birth care providers were recruited from four large cities in the Netherlands which have relatively high smoking rates (Rotterdam, the Hague, Utrecht, and Arnhem) [35]. The research team used public data to select eligible midwifery practices within or near socially disadvantaged city districts [36]. A list of eligible midwifery practices (*N* = 28) was made; these practices were contacted by telephone to invite one birth care provider employed in the practice to participate in an interview. Potential participants were invited to be interviewed at a time and location of their choice.

Of the 28 eligible midwifery practices, 13 practices did not respond to our request. Of the 15 practices that did respond, 8 practices declined participation, as they had other priorities (*n* = 6), were participating in other studies (*n* = 1), or had a lack of experience with smokers (*n* = 1). Seven practices from the 28 eligible midwifery practices responded positively to the request to have a birth care provider participate in an interview.

Simultaneously, convenience sampling was used to recruit birth care providers to participate in interviews. The study team invited 7 birth care providers with whom they had had previous contact, because they participated in an exploratory research for a smoking cessation intervention for pregnant women [37]. In this exploratory research, birth care providers shared their experiences on smoking cessation care for pregnant women in focus groups [results are not published]. The study team only invited birth care providers to participate in this study, who were not involved in the intervention study. The birth care providers were contacted by telephone or by email. All 7 birth care providers agreed to participate and were interviewed at a time and location of their choice. These birth care providers were located in the Hague (*n* = 2), Utrecht, Zeist, Gouda, Leiden and Zwolle.

### Data collection and setting

Thirteen interviews were conducted with 14 birth care providers. The interviews were conducted by two female researchers, working in the field of smoking cessation and trained in qualitative research methods (EW and LS). Face-to-face interviews were conducted between November 2017 and February 2018. The interviews took place at midwifery practices (*n* = 12 interviews with 13 birth care providers) or at the participant’s home (*n* = 1). One interview was conducted with two midwives simultaneously, both working at different midwifery practices. All interviews were conducted in Dutch. The interviews took 30–60 minutes (average: 40 minutes) and were recorded. The interviews were transcribed verbatim.

### Coding

All interviews were coded [38] using MAXQDA18. EW and LS familiarized themselves with the data by reading the transcripts. An inductive coding approach was applied where data-driven codes on the influence of the social circle on pregnant women and the barriers birth care providers perceive in discussing smoking cessation with the social circle of pregnant women were generated. After coding five interviews individually, EW and LS discussed their findings and developed a preliminary list of codes. After coding the following four interviews individually, EW and LS reviewed the codes and discussed the definitions of codes to determine agreement. The final list of codes was developed; EW went back to the previously coded interviews to include the new codes. EW coded the remaining four interviews with the final list of codes (see Additional File 2).

### Data analysis

Data were analyzed according to the principles of thematic analysis by Braun and Clarke, who define a theme as: *“A theme captures something important about the data in relation to the research question, and represents some level of patterned response or meaning within the data set”* [39]. After data familiarization and agreement between EW and LS on the initial coding process, codes were grouped in preliminary themes by EW. Comparison between coded data was used to identify new patterns from qualitative data and ensuring that the themes reflected these patterns. EW then summarized these preliminary themes into paragraphs for a Dutch-language report [40]. LS reviewed the preliminary themes for the Dutch-language report. Through discussion between EW and LS consensus was reached on the definitions and names of the themes. The paper was produced including only the themes related to the aim of this paper; see Additional File 3 for more information on the themes.

### Translation

Quotes were translated from Dutch to English by EW. A native English speaker (BJHW) assisted with translation of a selection of quotes.

## Results

### Participant details

Fourteen birth care providers were interviewed: twelve primary care midwives, one primary and secondary care midwife, and one OB-GYN. The birth care providers were all women and had at least 1 year experience working in prenatal care. The results were primarily derived from interviews with midwives; the term “midwives” will be used hereafter unless the data was from the interview with the OB-GYN.

### Results of the analysis

Three main themes were identified during data analysis. These themes were: (1) midwives’ experiences with assisting members of the social circle with cessation, (2) perceived barriers to discussing cessation with members of the social circle, and (3) midwives’ role in assisting the social circle with cessation.

#### Experiences with assisting members of the social circle with cessation

Midwives believed that a pregnant women’s social circle has a considerable influence on her smoking status:

*“They [the social circle] play an important role. What you often see is that if women smoke, their partners and their mothers smoke too. You rarely come across a woman who is the only one who smokes in a smoke-free social circle.”* (Midwife 1)

When it concerns the social circle of pregnant women, midwives come across partners of pregnant women more often during antenatal appointments than family members or friends.

*"Partners often attend the first consultation [with the midwife], but their attendance later in pregnancy varies. Some partners we never see and others come along every time. On average, they come along 3 or 4 times. [ … ] Sometimes a [grand] mother comes along, but not very often. We even less often see a friend. ”* (Midwife 6)

While midwives acknowledge that pregnant women who smoke are usually part of a social circle in which smoking is common, midwives noted that they do not commonly ask members of the pregnant woman’s social circle if they smoke:

*“When I ask a pregnant woman about smoking, it could be that her mother also spontaneously says something about smoking, but I am not going to ask mothers, sisters, or friends about their smoking.”* (Midwife 13)

When midwives reported when they talk about smoking with partners, this usually happens only once with limited or no follow-up:

*“I always mention in the first consultation that quitting smoking together is easier. If I am being honest, after that I let go of the partner a little bit and I focus on the pregnant woman. I mention it [smoking cessation] again to pregnant women, but I won’t mention it again to the partner.”* (Midwife 2)

Further, when midwives do talk about smoking with partners or members of the pregnant woman’s social circle, the focus may be limited to harm reduction. As the quote below reveals, the midwife views smoking cessation as not necessarily feasible:

*“Well, smoking outside is often the highest attainable [goal], because they have complicated lives and it [smoking] is just hard to deal with.”* (Midwife 11)

This view may influence how midwives address the smoking behavior of pregnant women’s partners.

#### Barriers to addressing smoking with members of the social circle

Midwives faced barriers to provide cessation support to the social circle of pregnant women. One barrier midwives perceived is the lack of a trusting relationship with the social circle of pregnant women:

*“I wouldn’t proactively ask a mother [of a pregnant woman] during a consultation if she smokes, even though I know it would be good to do. [ … ] I feel less connected to the pregnant woman’s mother than to her partner.”* (Midwife 2)

Moreover, midwives expressed that they do not want to risk their relationship with the pregnant woman by discussing smoking with her social circle:

*“The trusting relationship can be damaged by involving her mother. That’s why it’s such a big problem all around.”* (Midwife 5)

Concerns about raising the topic with members of the social circle of pregnant women were expressed by the interviewed midwives:

*“Sometimes it feels a little … how can I explain that … it feels a little like I’m interfering too much with them.”* (Midwife 3)

According to some midwives, partners do not want to talk about smoking or smoking cessation with them:

*“..I think that partners are less open to smoking cessation support [than pregnant women], because they don’t really see why a midwife should discuss this with them.”* (Midwife 2)

Midwives were concerned about giving unwanted smoking cessation advice to the members of the social circle. They were also concerned that the social circle do not think that midwives are the right professionals to receive cessation advice from.

Another barrier many midwives perceived is a lack of opportunity to discuss smoking:

*“We only see them [pregnant women and partners] for about seven months or so and after that it stops. That is a short amount of time. I’m in a practice with four midwives, so how many times do you see someone personally?”* (Midwife 11)

When a pregnant woman receives care at a group practice, she may see different midwives during her pregnancy. As partners and/or other members of a woman’s social circle do not attend every visit, the midwife has limited interactions with a pregnant woman’s friends, family, and partners and limited opportunities to address smoking.

Midwives stated that most partners had already made changes to their smoking:

*“You know, in general, of course that [quitting smoking] is discussed at some point during the pregnant woman’s care. In 9 out of 10 cases, a partner has already made changes and goes to the balcony or outside to smoke. If the pregnant woman is okay with that, who are we to say that it is not okay?”* (Midwife 9)

According to midwives, most partners believe that they are already doing enough by smoking outside, a belief which is echoed by the pregnant women themselves. As a result, some midwives did not encourage partners to make further changes by quitting smoking.

#### Midwives’ role in assisting the social circle with cessation

The midwives described being ambivalent about their responsibility to provide smoking cessation support to a pregnant woman’s social circle. When asked about the midwife’s role with regard to assisting the social circle with smoking cessation, one midwife stated:

*“Yes and no, I think our role as midwife is to take care of the health of the mother and child. The social circle can help, but I think it is their own responsibility too. [ … ] I am not sure if I am really responsible for that [smoking cessation support of the social circle].”* (Midwife 5)

However, other midwives noted that cessation advice to the social circle would be important to do as part of care for pregnant women:

*“On the one side it is [the role of a midwife], because I think that if the social circle is engaged, more pregnant women will successfully quit smoking. This is eventually our goal. On the other side, we must draw a line somewhere, also from a time/resources point of view.”* (Midwife 2)

Some midwives saw a limited role in engaging the social circle.

*“I think that it is the role of a midwife to get a picture [of the smoking behavior] of the social circle. I don’t think it is our role to mobilize the social circle in smoking cessation. I find it hard to tell if that fits our role.”* (Midwife 6)

Midwives stated that other healthcare professionals, such as General Practitioners (GPs) and youth health care providers, could play an important role in smoking cessation counselling to both partners and the extended social circle of pregnant women.

*“GPs have a more equal treatment relationship with both the partner and the pregnant woman [than a midwife]. I have a better relationship with the pregnant woman of course. This makes the GP more suitable to provide smoking cessation counselling to the social circle [than a midwife].”* (Midwife 2)

While midwives indicated that a GP could help pregnant women and her social circle with smoking cessation, actively referring smokers to smoking cessation services is often not done, in part due to a lack of knowledge:

*“For us it was a problem that we didn’t have a guide of social services, where we refer someone if we want to refer them [pregnant woman and social circle]: what are the costs, what is useful and what is not?”* (Midwife 6)

In general, most midwives agree that they have to play some kind of role in smoking cessation counselling for the social circle, albeit a very limited one.

*“As a midwife my initial focus is on my important task of taking care of the baby and the mother. Of course, this [the social circle] is also included in this, but still I find it more important to focus on the pregnant woman than on her social circle.”* (Midwife 8)

## Discussion

The interview data shows that midwives acknowledged the importance of a pregnant women’s social circle when it comes to smoking cessation. However, while they found it important, the interviewed midwives noted that they don’t commonly provide smoking cessation support to members in the social circle.

In the Netherlands, primary care midwives are required by the Healthcare Inspectorate to counsel pregnant women and their partners to quit or reduce smoking by using the V-MIS protocol [28]. While partners are specifically mentioned in this protocol, other members of pregnant women’s social circle are not included. Absence of clear guidelines and protocols could influence the provision of effective smoking cessation advice to the pregnant women’s social circle [41]. The interviews showed that midwives were often ambivalent about their responsibility to provide smoking cessation support to a pregnant woman’s social circle. This ambivalence may have influenced the midwives’ interactions with the social circle.

Midwives encountered a variety of barriers when addressing smoking with members of a pregnant woman’s social circle. One such barrier was the fear of jeopardizing their relationship with the pregnant woman when they discuss smoking with her social circle. Having a trusting relationship with pregnant women was seen as needed for addressing tobacco use and exposure; previous studies found that midwives were concerned about adversely affecting the relationship they have with pregnant women by (repeatedly) asking about smoking [19, 42]. In addition, the interviewed midwives found it challenging to discuss smoking cessation with members of the social circle, as they felt less connected to them than to pregnant women. Research conducted in the Netherlands on nurses working within a preventive care program for disadvantaged young women during and after pregnancy (2019) [43] found that the ability to build on a trusting relationship with pregnant women was seen as useful for discussing smoking. As seen in our data and in the literature, addressing smoking and discussing smoking cessation is affected by having and keeping a trusting relationship with the pregnant woman and her social circle.

Midwives found it challenging to motivate partners to quit smoking when they had already taken steps to prevent exposing pregnant women to tobacco smoke. A study by Gage et al. [44] (2011) found that partners of pregnant women would rather reduce their smoking or smoke outside than quit smoking completely. Our data collection showed that, according to midwives, pregnant women and their partners believed that smoking outside was a legitimate way to reduce the risk and harms from exposure to second and thirdhand smoke, with complete smoking cessation by the partner seen as unnecessary. Some midwives in this study also believed that harm reduction, mainly smoking outside, is the highest attainable achievement a pregnant women’s social circle could reach. This may have influenced why and how midwives didn’t discuss quitting smoking completely with partners. However, it is important that partners quit smoking completely because women are less likely to quit themselves if their partner smokes [45] and smoking outside does not completely prevent second and thirdhand smoke exposure [46].

A study on the role of midwives and gynecologists in smoking cessation care of pregnant women in Belgium (2015) found that healthcare professionals saw their role as limited to asking about smoking, providing brief advice, determining the readiness to quit, and referring clients to specialized cessation counseling [41]. The interviewed midwives in the current study also saw a role for other healthcare professionals, especially GPs, in helping the social circle of pregnant women quit. In the Netherlands, health insurance is compulsory; in 2016, less than 0.2% of the population was uninsured [47]. Since 2020, health insurance covers one primary care smoking cessation program per year [48]. Referring to other healthcare professionals for (more intensive) smoking cessation counselling is part of the V-MIS protocol [27]. However, findings from the current study show that referring smokers for more intensive cessation counselling is often not done and referral options were not well known by midwives.

### Limitations

This study has a few limitations. Birth care providers were self-selected, which could indicate an interest in smoking cessation. A more representative group of birth care providers may have different insights and experiences. Our study only took into account the perspectives of Dutch birth care professionals in providing smoking cessation advice to the social circle. Future research should explore the views of the social circle in receiving smoking cessation from birth care providers.

## Conclusions

This study provides insights in why midwives in the Netherlands may be reluctant to actively provide smoking cessation advice to the social circle of pregnant women. The interviews showed that midwives can be ambivalent about their responsibility to provide smoking cessation support to a pregnant woman’s social circle, which may influence the interaction they have with the social circle. In addition, midwives may have barriers to discussing smoking cessation with the social circle of pregnant women, such as a lack of a trusting relationship with the social circle, concerns about raising the topic, and giving unwanted advice on cessation to members of the social circle and a lack of opportunity to discuss smoking.

### Practical implications

Pregnancy can be a teachable moment for smoking cessation for members of pregnant women’s social circle [23–25]. Partners of pregnant women can be engaged in smoking cessation efforts by advising them on the risks of secondhand smoke and on quitting smoking [8]. To overcome barriers such as damaging their trusting relationship with pregnant women, educational programs or new modules for existing program could be used to improve skills related to discussing smoking with the social circle of pregnant women [19, 37].

Clear guidelines and protocols on the role of birth care providers in providing smoking cessation support to the social circle could help midwives overcome ambivalence that they might have. While midwives have a unique role in providing smoking cessation advice and support to pregnant women and members of pregnant women’s social circle, the smoking cessation advice, support, and care that they can provide is limited by time and opportunities to interact with the social circle and by their role and skills, which may not be the best suited for intensive smoking cessation support for members of the social circle. To that end, it is crucial that midwives know where and how to refer smokers to other healthcare professionals for more intensive smoking cessation care [41]. The development and implementation of care pathways in primary care midwifery practices could contribute to a better referral to other healthcare professionals [49].

## Supplementary Information

**Additional file 1.** Interview guide for birth care providers.

**Additional file 2.** Coding scheme.

**Additional file 3.** Identification of themes.

## Data Availability

The datasets generated and/or analyzed during the current study are not publicly available because it was a qualitative study but are available from the corresponding author in an anonymized form if requested.

## Abbreviations

GP: General Practitioner
OB-GYN: Obstetrician-gynecologist
V-MIS: Minimal Intervention Smoking Cessation Strategy for Midwifery Practices (in Dutch: Minimale Interventiestrategie Stoppen met Roken voor de Verloskundigenpraktijk)
