# Supplementary material for: “If the social circle is engaged, more pregnant women will successfully quit smoking”: a qualitative study of the experiences of midwives in the Netherlands with smoking cessation care
Source: BMC Health Serv Res. 2022 Aug 31;22:1106. doi: 10.1186/s12913-022-08472-7 (PMC9429426; doi:10.1186/s12913-022-08472-7)
Supplement: Supplementary file 1 — Additional file 1. Interview guide for birth care providers. [file 12913_2022_8472_MOESM1_ESM.docx]

**Additional file 1. Interview guide for birth care providers**

Thank you for participating in this study. I am [name researcher] and I work as a researcher at [name institute] on the topic of tobacco control. In this study we would like to focus on the social circle of pregnant women, such as partners, mothers or other relatives and friends, who play an important part in the lives of pregnant women. We aim to gain more insights in how smokers from the social circle of pregnant women can be supported to stop smoking. We will interview (former) smokers from the social circle of pregnant women and birth care providers such as midwifes and gynecologists. In this interview I would like to discuss your experiences with the smoking social circle of pregnant women, the barriers you come across and your role in involving the social circle in smoking cessation.

Participation in this study is anonymous. To make sure I will be able to use all things that we talked about during this interview I would like to make an audio recording. Are you okay with this? The audio recordings will be analyzed anonymously and we will not share your personal details with third parties. If you want to withdraw your participation from this study, you can do this without telling us why. Do you have any questions before we start the interview? If you consent to participate, could you please sign this consent form? [start recording]

**Interview**

- Can you tell me something about yourself?

Introducing question:

- What comes into your mind when you think about smoking cessation and the social circle of pregnant women?

Consultation with birth care providers:

- Do partners come along with pregnant women to a consultation with you? How often?
- Do other persons from the social circle of pregnant women come along to a consultation with you? How often?

Experiences with a smoking social environment:

- Do you often encounter pregnant women who smoke or of which the social circle smokes?
- Do you use a protocol for smoking cessation counselling?
- Do you involve partners of pregnant women in smoking cessation? In which way?
- What do you advice to smoking partners, relatives and friends of pregnant women?
- Do you use materials for smoking cessation? Also for the social circle of pregnant women? What do you think of these materials?
- Are there any strategies you use that work to reach the social circle of pregnant women?
- What do you think is the best way to reach the social circle of pregnant women?
- How do you see your role in involving the social circle of pregnant women in smoking cessation support? Do you see a role for another care giver in that?

Perceived barriers in smoking cessation support:

- Do you encounter barriers in involving partners in smoking cessation? Which?

Desired changes in smoking cessation support:

- What should change concerning the support of the social circle of pregnant women in smoking cessation?
- What do you think of the existing materials on smoking cessation? Would you change something?
