# Supplementary material for: “If the social circle is engaged, more pregnant women will successfully quit smoking”: a qualitative study of the experiences of midwives in the Netherlands with smoking cessation care
Source: BMC Health Serv Res. 2022 Aug 31;22:1106. doi: 10.1186/s12913-022-08472-7 (PMC9429426; doi:10.1186/s12913-022-08472-7)
Supplement: Supplementary file 3 — Additional file 3. Identification of themes. [file 12913_2022_8472_MOESM3_ESM.docx]

**Additional file 3. Identification of themes**

Table 2. Coding scheme

| Theme 1: Experiences with assisting members of the social circle with cessation |
| --- |
| Social circle’s influence |
| Advice to partners |
| Advice to extended social circle |
| No follow-up on advice |
| Advice to smoke outside |
| Theme 2: Perceived barriers to discussing cessation with members of the social circle |
| Hard to reach |
| Lack of contact with social circle |
| Lack of health care continuity |
| Lack of skills to motivate |
| Concerns in raising the topic |
| Trusting relationship |
| Lack of motivation to quit |
| Low SES |
| Time constraints |
| Socially desirable answers |
| Theme 3: Birth care providers’ role in assisting the social circle with cessation |
| Responsibility |
| Focus on women and child |
| Ambivalence of role perception |
| Role other health care professionals |
| Referral |
